# Six-month randomized, placebo controlled trial of synbiotic supplementation in women with polycystic ovary syndrome undergoing lifestyle modifications

**DOI:** 10.1007/s00404-024-07833-3

**Published:** 2024-12-05

**Authors:** Izabela Chudzicka-Strugała, Anna Kubiak, Beata Banaszewska, Ewa Wysocka, Barbara Zwozdziak, Martyna Siakowska, Leszek Pawelczyk, Antoni J. Duleba

**Affiliations:** 1 Department of Medical Microbiology, Poznan University of Medical Sciences, 60-535 Poznan, Poland; 2 Division of Infertility and Reproductive Endocrinology, Department of Gynecology, Obstetrics and Gynecological Oncology, Poznan University of Medical Sciences, 60-535 Poznan, Poland; 3 Department of Laboratory Diagnostics, Poznan University of Medical Sciences, 60-569 Poznan, Poland; 4 Division of Reproductive Endocrinology and Infertility, Department of Obstetrics, Gynecology and Reproductive Sciences, University of California San Diego, 9500 Gilman Drive, 0633, San Diego, CA 92093-0633 USA

**Keywords:** Polycystic ovary syndrome, Synbiotic, Diet, Testosterone

## Abstract

**Purpose:**

To determine whether long-term administration of synbiotics affects clinical, endocrine and metabolic aspects of polycystic ovary syndrome (PCOS) in overweight and obese subjects undergoing intensive lifestyle modifications.

**Methods:**

During six-month trial, all subjects underwent intensive lifestyle modifications (diet and exercise). The subjects were randomized (1:1) to receive synbiotic supplementation (Synbiotic Group) or placebo (Placebo Group).

**Results:**

Subjects in the Placebo Group and the Synbiotic Group experienced significant reduction of BMI (− 8% and − 11%, respectively; both at *P* < 0.0001) and body fat percentage (− 11% and − 14%, respectively; both at *P* < 0.0001). These effects were statistically comparable for both groups. Total testosterone was not significantly changed in the Placebo Group (− 5%, *P* = 0.41) while it greatly declined in the Synbiotic Group (− 40%; *P* < 0.0001); the difference between these groups was significant (*P* = 0.0002). Synbiotic supplementation was superior to placebo in reducing LH (− 21%; *P* = 0.047), total cholesterol (− 6%; *P* = 0.002), low-density lipoprotein cholesterol (− 6%; *P* = 0.044), triglycerides (− 29%; *P* = 0.049), LPS (− 23%; *P* = 0.001) and LPS-binding protein (− 21%; *P* = 0.001).

**Conclusions:**

Synbiotic supplementation led to a marked improvement of several key clinical and laboratory aspects of PCOS including an improvement of hyperandrogenism, lipid profile, and markers of endotoxemia.

**Trial registration:**

Clinical Trial Registration Number: NCT03325023 (URL, clinicaltrials.gov; date of registration 10/26/2017).

## Introduction

One of the greatest challenges facing medicine is the development of effective weight loss programs for overweight and obese patients. The obesity epidemic in the United States is worsening; recent surveys estimated that approximately 42% of adults are obese and another 32% are overweight [18, 42]. Polycystic ovary syndrome (PCOS) is the most common endocrinopathy among women in reproductive age [34] and often associated with excessive weight with a pooled estimated prevalence of obesity at 61% [24]. While excessive weight itself may not be a cause of PCOS, it clearly contributes to a worsened phenotype characterized by a wide range of adverse cardiometabolic and endocrine features, including insulin resistance associated with compensatory hyperinsulinemia, dyslipidemia, and systemic inflammation [1, 16, 19, 20].

Growing evidence points to significant benefits of reducing weight through healthy lifestyle changes including diet and exercise as presented in recent evidence-based guidelines discussing women with PCOS [22, 30]. Notably, lifestyle modifications alone (exercise and/or diet) may result in a marked weight loss but, at best, lead to only a modest effect on testosterone level, parameters of insulin sensitivity, and lipid profile [10, 12, 23, 37]. Another non-surgical approach to the treatment of obesity and its endocrine and metabolic sequelae targets gut microbiota. The underlying concept of such therapy proposes that an adverse microbiome (i.e., dysbiosis) is associated with obesity and may lead to endotoxemia (presence of lipopolysaccharides-LPS in the bloodstream), resulting in systemic inflammation, insulin resistance, and even excessive ovarian production of androgens in women with PCOS [5, 7, 8, 39, 40]. Indeed, our previous studies have demonstrated that markers of endotoxemia, such as serum LPS and LPS-binding protein, are elevated in women with PCOS and that LPS may stimulate androgen production by ovarian tissues [3, 14]. Modification of gut microbiome may be accomplished by supplementing diet with probiotics (bacteria associated with health benefits) and prebiotics (non-viable food components affecting gut flora). In obese subjects, the use of probiotics and prebiotics has been shown to improve gut microbiome and may lead to a reduction of weight and an improvement of metabolic profile [32, 36, 41]. Synbiotics are a preparation containing a combination of probiotics and prebiotics. These supplements have been shown to increase the relative abundance of gut bacteria associated with health benefits and decrease the abundance of bacteria associated with inflammation and obesity [36].

In view of the above considerations and the knowledge gap regarding the effects of synbiotics in women with PCOS, the present study was designed to evaluate effects of intensive lifestyle modifications alone (Placebo Group) or in combination with synbiotics supplementation (Synbiotic Group) in overweight and obese women with PCOS. Preliminary findings after three months revealed that diet and exercise led to significant reduction of BMI while the addition of synbiotics resulted in a significant reduction of total testosterone [10]. However, after three months, synbiotics did not have significant effect on other important aspects of PCOS, such as LH and lipid profile. This report describes the final findings of a six-month randomized trial. As demonstrated below, after six months of treatment, women receiving synbiotics experienced additional improvements in several parameters of metabolic health; furthermore, symbiotic supplementation led to significant reduction in markers of endotoxemia.

## Materials and methods

### Subjects

**Fig. 1:**
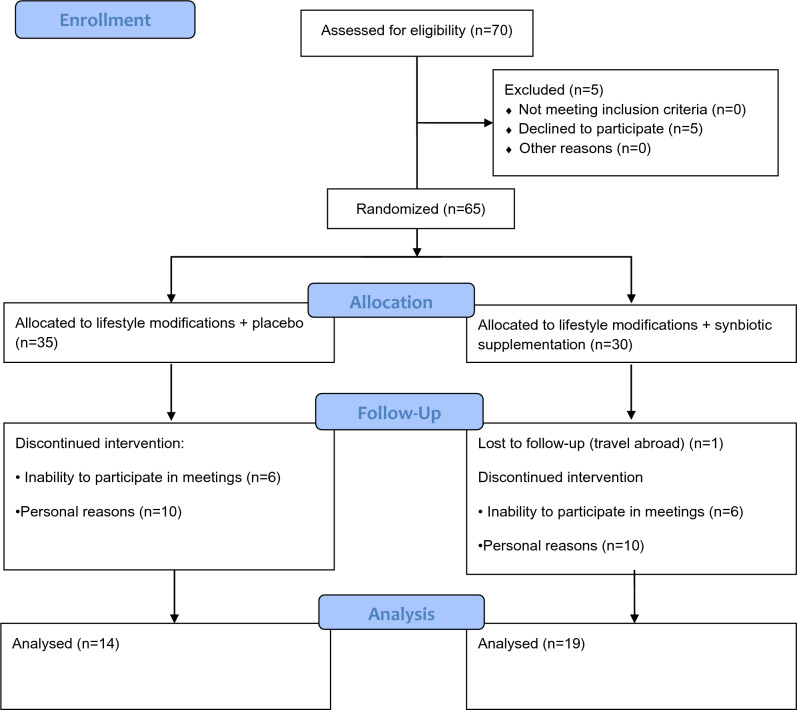
Diagram summarizing the design, enrollment, randomization, and follow-up of subjects in this study (CONSORT flow diagram)

The study was carried out at the Reproductive Endocrinology and Infertility Clinical Services at Poznan University of Medical Sciences. Seventy overweight or obese (BMI > 25) women with PCOS were invited to participate in this trial. Sixty-five women consented and were randomized as presented in Fig. 1. PCOS was defined according to the criteria established by the Rotterdam consensus in the presence of at least two of the following three features: (1) clinical or chemical hyperandrogenism; (2) oligo- or amenorrhea; and/or (3) polycystic ovaries as determined by transvaginal ultrasound [17]. Other endocrinopathies, such as congenital adrenal hyperplasia, thyroid disease, hyperprolactinemia, Cushing disease, and diabetes mellitus, were excluded. Furthermore, for the last two months before commencing this trial, none of the subjects used any hormonal therapies, metformin, antibiotics, laxatives, dietary supplements for weight loss, probiotics, or synbiotics. The study was approved by the Institutional Review Board at the Poznan University of Medical Sciences and written consent was obtained from all participants. The study was registered at www.clinicaltrials.gov with the identifier *NCT03325023*.

### Procedures

Consenting subjects were randomized into two groups: the Placebo Group and the Synbiotic Group. Randomization was carried out using a 1:1 allocation ratio with blocks of four. Patient allocation was obtained using GraphPad QuickCalcs (GraphPad Software Inc., La Jolla, CA). Patients and investigators were blinded to treatment. Both groups received identical intensive lifestyle modifications consisting of closely monitored low-carbohydrate and low-fat diet in combination with an exercise regimen. Diet included restricting caloric intake to 1400–1800 kcal/day based on body composition analysis as well as individualized advice regarding the selection of food composition, exclusion of alcohol, reduction of salt intake, and avoidance of fast-food. Diet consisted of 40–45% of carbohydrates, 20–25% proteins and 25–35% fat. The exercise regimen included walks for 30–40 min every day. To maximize compliance, all subjects were followed up by a dietician in person every two to three weeks. The study was initiated in 2017 and completed in 2021.

Women in the Placebo Group received four capsules of placebo daily while those in the Synbiotic Group received four capsules of synbiotic supplementation daily. Synbiotics (SANPROBI Super Formula) consisted of the following probiotics: *Bifidobacterium lactis (W51 and W52), Lactobacillus acidophilus (W22), Lactobacillus paracasei (W20), Lactobacillus plantarum (W21, Lactobacillus salivarius (W24), and Lactobacillus lactis (W19)* as well as prebiotics fructooligosaccharides and inulin. The selection of the above preparation was based on the presence of bacteria species and strains considered to be beneficial to metabolism and inclusion of oligosaccharides and inulin due to their effects on gut microbiota and anti-inflammatory properties [28, 33].

Primary end-points were changes in BMI, body composition, and total testosterone. Evaluations were carried out at baseline during the follicular phase of a natural cycle or after medroxyprogesterone-induced menses; final evaluations were carried out after six months of treatment. Clinical assessments included determinations of weight/body mass index (BMI), fat content (Tanita multifrequency body composition analyzer; Tokyo, Japan), circumference of waist, hip and thigh, and hirsutism (Ferriman and Gallwey score). Transvaginal ultrasound evaluations of both ovaries were performed using Aloka ProSound α7 (Aloka Co, Ltd, Tokyo, Japan). Ovarian volume was determined using the prolate ellipsoid formula.

Blood was collected after an overnight fast, and serum specimens were stored at – 70 °C until analysis. A two-hour 75 g oral glucose tolerance test was performed with determinations of glucose and insulin in the fasting state followed by testing at 30, 60, 90, and 120 min after glucose ingestion. Glucose was determined using the enzymatic reference method with hexokinase (Cobas c501, Roche Diagnostics Polska, ROCHE Holding AG, Basel, Switzerland). Insulin, total testosterone, LH, FSH, SHBG, and DHEAS were determined using electrochemiluminescence assays (Cobas c501, Roche Diagnostics Polska, ROCHE Holding AG, Basel, Switzerland). The insulin sensitivity index (ISI) was determined using glucose and insulin levels obtained during an oral glucose tolerance test as described by Matsuda and DeFronzo: Insulin Sensitivity Index = (10,000/square root of [(fasting glucose x fasting insulin) x (mean glucose x mean insulin during oral glucose tolerance test)] [29]. Total cholesterol and triglycerides were determined using enzymatic colorimetric assays (Cobas c501, Roche Diagnostics Polska, ROCHE Holding AG, Basel, Switzerland). High-density lipoprotein (HDL) was separated by precipitating apolipoprotein-B (Roche Polska sp z o.o., Warsaw, Poland. Low-density lipoprotein cholesterol (LDL-C) was calculated using the Friedwald formula.

### Statistical analysis

Analysis was performed using JMP pro 16 statistical software (SAS Institute, Cary, NC). *P*-values < 0.05 were considered significant. Comparisons of continuous variables between groups were performed using an unpaired *t*-test or, when appropriate, the Mann–Whitney *U* test. Comparisons of baseline and follow-up values were performed using paired *t*-test, or in the absence of a normal distribution (tested by Shapiro–Wilk test), Box–Cox transformations or non-parametric testing (Wilcoxon signed-rank) was carried out. Categorical variables were compared using Fisher’s exact test. Multiple regression modeling was performed using both forward and backward stepwise approach evaluating all variables listed in Table 2. Power analysis revealed that a detection of the effect size of 1 would require at least 11 subjects in each group (power of 0.8 and alpha error of 0.05).

## Results

### Baseline characteristics of study participants

Figure 1 (CONSORT flow diagram) summarizes enrollment, randomization, and follow-up of participants in this study up to the endpoint at 6 months. The trial was completed by 33/65 (51%) of enrolled subjects; there was a trend toward a greater retention of subjects in the Synbiotic Group (19/30; 63%) than in the Placebo Group (14/35; 40%); however, this difference did not reach statistical significance (*P* = 0.06). At baseline, clinical, endocrine, and metabolic characteristics of women who completed the study in both groups were comparable with the exception of a significantly higher LPS:HDL ratio (one of the markers of endotoxemia) in the Synbiotic Group in comparison to the Placebo Group (Table 1).

**Table 1:** Baseline values of subjects who completed the 6 months trial (mean ± SEM)

Variable	Placebo Group *N* = 14	Synbiotic Group *N* = 19	*P*-value
Age	29.9 ± 1.5	31.2 ± 0.8	0.41
Weight (kg)	95.4 ± 4.3	92.9 ± 2.6	0.61
BMI (kg/m^2^)	34.2 ± 1.5	33.5 ± 1.0	0.67
Body fat (%)	40.0 ± 1.3	40.6 ± 1.1	0.73
Waist circumference (cm)	109 ± 3.3	109 ± 2.1	0.98
Hip circumference (cm)	108 ± 3.1	105 ± 1.7	0.34
Thigh circumference (cm)	64.2 ± 1.4	65.3 ± 1.3	0.57
WHR	0.99 ± 0.02	1.04 ± 0.02	0.10
Hirsutism (Ferriman/Gallwey score)	8.6 ± 1.3	10.5 ± 1.0	0.27
Ovarian volume (both ovaries, mL)	20.8 ± 1.8	21.0 ± 1.3	0.92
Total testosterone (ng/mL)	0.60 ± 0.03	0.57 ± 0.04	0.48
DHEAS (µmol/mL)	8.19 ± 0.85	7.38 ± 0.84	0.51
SHBG (nmol/L)	36.3 ± 3.7	42.0 ± 5.2	0.42
LH (mIU/mL)	8.6 ± 1.5	9.7 ± 1.0	0.56
FSH (mIU/mL)	5.0 ± 0.4	4.4 ± 0.5	0.40
LH:FSH ratio	1.76 ± 0.33	2.47 ± 0.28	0.11
Fasting insulin (µU/mL)	14.3 ± 1.9	14.3 ± 1.3	1.00
Fasting glucose (mg/dL)	89.6 ± 1.9	92.5 ± 2.0	0.31
ISI (Matsuda)	3.9 ± 0.8	3.0 ± 0.3	0.27
Total cholesterol (mg/dL)	166 ± 10	183 ± 10	0.25
LDL cholesterol (mg/dL)	90 ± 11	106 ± 8	0.22
HDL cholesterol (mg/dL)	58 ± 7	53 ± 2	0.44
Triglycerides (mg/dL)	94 ± 11	122 ± 14	0.13
LPS (EU/mL)	2.66 ± 0.43	3.42 ± 0.28	0.15
LPS:HDL ratio	0.043 ± 0.008	0.07 ± 0.007	0.02
LBP (µg/mL)	29.6 ± 4.8	36.1 ± 3.0	0.23

### Effects of treatments at six months

Table 2 summarizes the outcomes of this study. After six months of intensive lifestyle modification, highly significant declines of BMI and body fat percentage were observed in both study groups. All participants in both study groups lost weight. In the Placebo Group, this weight loss ranged from 2.8 kg to 13 kg while in the Synbiotic Group the weight loss ranged from 2.8 kg to 20.3 kg. Trends toward greater effects on these parameters among users of Synbiotics did not reach statistical significance. Both groups experienced a significant reduction in waist, hip and thigh circumferences. Subjects in the Synbiotic Group achieved a statistically greater decrease in waist circumference (by 13% vs. 9%; *P* = 0.03) and thigh circumference (by 10% vs. 6%; *P* = 0.004) in comparison to those in the Placebo Group. Both groups experienced a significant decrease in hirsutism. Decreases in ovarian volume appeared to be numerically comparable in both groups (*P* = 0.92). However, this effect reached statistical significance only in the Synbiotic group.

**Table 2:** The effect of lifestyle and diet ± placebo or Synbiotic treatment at 6 months. Statistically significant (*P*<0.05) differences between groups are presented as bold numbers

	Placebo Group		Synbiotic Group		
Effect	Change at 6 months	*P*-value vs. baseline	Change at 6 months	*P*-value vs. baseline	*P*-value (between groups)
Weight (kg)	− 7.78 ± 0.91 (− 8%)	< 0.0001	− 10.10 ± 1.17 (− 11%)	< 0.0001	0.30
BMI (kg/m^2^)	− 2.79 ± 0.31 (− 8%)	< 0.0001	− 3.63 ± 0.42 (− 11%)	< 0.0001	0.17
Body fat (%)	− 4.4 ± 0.8 (− 11%)	< 0.0001	− 5.7 ± 0.7 (− 14%)	< 0.0001	0.25
Waist circumference (cm)	− 10.0 ± 1.7 (− 9%)	< 0.0001	− 14.3 ± 1.1 (− 13%)	< 0.0001	**0.03**
Hip circumference (cm)	− 8.1 ± 1.3 (− 8%)	0.0007	− 10.1 ± 1.2 (10%)	< 0.0001	0.28
Thigh circumference (cm)	− 4.0 ± 0.5 (− 6%)	< 0.0001	− 6.3 ± 0.5 (− 10%)	< 0.0001	**0.004**
WHR	0.00 ± 0.02 (0%)	0.87	− 0.04 ± 0.01 (− 4%)	0.008	0.19
Hirsutism (Ferriman/Gallwey score)	− 0.5 ± 0.2 (− 6%)	0.01	− 1.1 ± 0.4 (− 10%)	0.02	0.30
Ovarian volume (both ovaries, mL)	− 3.6 ± 1.8 (− 17%)	0.07	− 3.4 ± 1.0 (− 16%)	0.003	0.92
Total testosterone (ng/mL)	− 0.03 ± 0.04 (− 5%)	0.41	− 0.23 ± 0.03 (− 40%)	< 0.0001	**0.0002**
DHEAS (µmol/ml)	0.73 ± 0.83 (9%)	0.39	0.62 ± 0.37 (8%)	0.11	0.90
SHBG (nmol/L)	10.3 ± 3.7 (28%)	0.002	2.7 ± 2.3 (6%)	0.25	0.07
LH (mIU/mL)	1.6 ± 1.7 (19%)	0.36	− 2.0 ± 0.9 (− 21%)	0.03	**0.047**
FSH (mIU/mL)	0.1 ± 0.6 (2%)	0.84	1.2 ± 0.7 (27%)	0.09	0.23
LH:FSH ratio	0.32 ± 0.37 (18%)	0.40	− 1.05 ± 0.29 (− 43%)	0.002	**0.004**
Fasting insulin (µU/mL)	− 1.6 ± 1.1 (− 11%)	0.17	− 5.1 ± 1.5 (− 36%)	0.004	0.10
Fasting glucose (mg/dL)	1.5 ± 2.3 (2%)	0.53	− 2.4 ± 1.5 (− 3%)	0.12	0.15
ISI (Matsuda)	0.21 ± 0.74 (5%)	0.77	2.11 ± 0.66 (70%)	0.005	0.07
Total cholesterol (mg/dL)	12.7 ± 3.9 (8%)	0.009	− 10.7 ± 5.6 (− 6%)	0.08	**0.002**
LDL cholesterol (mg/dL)	16.4 ± 9.0 (18%)	0.10	− 6.2 ± 5.0 (− 6%)	0.23	**0.044**
HDL cholesterol (mg/dL)	− 0.2 ± 6.8 (0%)	0.98	1.9 ± 1.9 (4%)	0.31	0.77
Triglycerides (mg/dL)	− 10.6 ± 4.6 (− 11%)	0.043	− 35.4 ± 10.8 (− 29%)	0.006	**0.049**
LPS (EU/mL)	− 0.23 ± 0.09 (− 9%)	0.03	− 0.80 ± 0.11 (− 23%)	< 0.0001	**0.001**
LPS:HDL ratio	− 0.0007 ± 0.005 (− 1%)	0.90	− 0.018 ± 0.003 (− 25%)	< 0.0001	**0.001**
LBP (µg/mL)	− 3.1 ± 1.6 (− 10%)	0.09	− 7.5 ± 0.8 (− 21%)	< 0.0001	**0.004**

One highly significant difference between the study groups (*P* = 0.0002) was observed regarding the change of total testosterone, which declined by 40% (*P* < 0.0001) in the Synbiotic Group versus 5% (*P* = 0.41) in the Placebo Group. Testosterone decline was observed in 100% of women in the Synbiotic Group and in 64% of women in the Placebo Group; this difference in the proportions was also significant (*P* = 0.008). Multiple regression analyses identified two independent predictors of decline of testosterone: the use of synbiotics and the baseline total ovarian volume (Table 3); these parameters explained 43% of the variance in this statistical model. These findings demonstrate that a higher baseline volume of both ovaries correlated with a greater decline of total testosterone.

**Table 3:** Multiple linear regression model predicting the decline of total testosterone level

Term	Estimate	*t*-ratio	*P*-value
Intercept	0.05	0.63	0.53
Group (Placebo vs. Synbiotic)	0.10	4.44	0.0001
Ovarian volume at baseline	− 0.01	− 2.39	0.02

Adjusted R-squared: 0.43

Final results of multiple regression modeling (forward and backward stepwise approach evaluating all variables listed in Table 2)

Interesting and significant differences between the study groups were also observed regarding the effects on gonadotropins, insulin, lipids, and markers of endotoxemia. LH and LH:FSH ratio significantly declined in the Synbiotic Group and slightly, but not significantly, increased in the Placebo Group; the differences of these effects between the groups were significant. In the Synbiotic Group, significant effects were observed in the level of fasting insulin (decline) and ISI (increase); in contrast, no significant effects on these parameters were observed in the Placebo Group. There were significant differences between the groups regarding total cholesterol, LDL cholesterol, and triglycerides, whereby the Synbiotic Group had markedly better lipid profiles. Finally, there were highly significant differences between the groups in all three markers of endotoxemia, whereby decreases of LPS, LPS:HDL ratio, and LBP were much greater in the Synbiotic Group.

## Discussion

Findings of this study demonstrate significant and diverse long-term effects of synbiotic supplementation superimposed on the impact of intensive lifestyle modifications on clinical, endocrine, and metabolic aspects of PCOS in overweight and obese women. Six months of dietary interventions and exercise resulted in weight loss and a reduction of body fat in association with decreases in waist, hip, and thigh circumferences, but there was no change in waist-to-hip ratio (WHR). The addition of synbiotics led to a greater reduction in waist than hip circumference and hence a significant decrease of WHR. This observation is particularly relevant since high waist circumference is specifically associated with adverse cardiovascular risk profile [35].

Surprisingly, lifestyle modifications alone had no significant impact on key reproductive-endocrine features of PCOS: high testosterone, and elevated LH:FSH ratio. In contrast, the addition of synbiotics led to a large (40%) reduction of total testosterone level, as well as decreased LH and LH:FSH ratio; these observations underscore value of synbiotics as an effective treatment of reproductive derangements in obese women with PCOS.

Another important feature of PCOS is the dysregulation of glucose–insulin homeostasis leading to insulin resistance, compensatory hyperinsulinemia, and ultimately a greatly increased risk of developing of type 2 diabetes [2, 13, 21]. In the present study, diet and exercise had no significant effect on glucose and insulin while the supplementation with synbiotics resulted in a highly significant reduction in fasting insulin and an increase in insulin sensitivity.

PCOS is also associated with an adverse lipid profile, especially reduced HDL cholesterol and elevated triglycerides [43, 44]. Unexpectedly, six months of intensive lifestyle modifications alone led to a significant increase in total cholesterol, a trend toward higher LDL cholesterol, and no effect on HDL; these observations contrast with reports by others, whereby weight loss of 5–10% in women (not PCOS) led to significant reductions of total cholesterol and LDL cholesterol [6]. The reasons for this discrepancy are not readily apparent and may be related to differences in subjects’ age and specifics of diet/lifestyle modifications, as well as a difference in studied populations, whereby women with and without PCOS may differ with regard to lipid metabolism. On the other hand, both the present study and that of Brown et al. revealed a consistent and significant reduction of triglycerides [6].

In the present study, synbiotic supplementation had a beneficial effect on lipid profile, whereby after six months, women in the Synbiotic Group, when compared to the Placebo Group, had significantly lower total cholesterol, LDL cholesterol, and triglycerides. These observations are consistent with other reports evaluating the effects of probiotics and synbiotics on lipids [15] and indicate that weight loss alone may not be sufficient to improve some important cardiovascular risk factors.

Previously, we reported interim findings of this study after three months of interventions [10] demonstrating that synbiotic supplementation potentiated effects of diet and exercise, including improvement in weight, reduction of waist circumference, and a decrease of total testosterone. In the present report, after six months of interventions, we describe several novel additional long-term beneficial effects of synbiotics: significant reduction of LH, LH:FSH ratio, fasting insulin, ISI, and an improved lipid profile. Furthermore, now we have evidence that synbiotics improve key markers of endotoxemia: LPS, LPS:HDL ratio, and LBP, providing a potential mechanistic explanation of our observations. Subjects of the present study had two important risk factors for adverse gut microbiome (i.e., dysbiosis): obesity and PCOS [25, 26, 38, 45]. It is well-recognized that gut dysbiosis leads to an increased translocation of LPS into the circulation, contributing to systemic inflammation and adverse effects on female reproductive functions; probiotics/synbiotics may alleviate dysbiosis and its consequences [4, 9, 27].

One of the most significant and clinically important effects of synbiotic supplementation in this study was a 40% decrease in testosterone level. We postulate that this effect may be due to both direct and indirect effects of an improved microbiome and a reduction of circulating LPS. We previously reported that LPS directly stimulates androgen production by ovarian theca-interstitial cells [14]; thus, the presently observed decrease in testosterone may be due to a decrease of this stimulatory effect on theca cells. It is also likely that the decrease of LPS may have indirect effects on androgen production by attenuating two well-recognized inducers of androgen synthesis: LH and insulin.

An important limitation of the present study pertains to the high drop-out rate from the program. Notably, our drop-out rate of 49% was comparable to previous reports describing up to 60–80% attrition rate in interventional studies of treatments of obesity [11, 31]. A trend toward lower drop-out rate among the subjects in the Synbiotic Group may reflect a significantly greater weight loss at three months of this trial [10]; indeed, a study by Colombo et al. indicated that initial treatment response contributed to the adherence to the weight loss program [11]. Another limitation in this report is the absence of data on the effects of lifestyle modifications and synbiotics on gut flora; indeed, further studies of this subject are urgently needed. Notably, it is possible that the effects of synbiotics may be related to actions of prebiotics such as inulin.

In conclusion, the present study provides new evidence that a combination of synbiotic supplementation and intensive lifestyle modifications delivers several long-term beneficial effects in women with PCOS, including improvement of hyperandrogenism and amelioration of several cardiovascular risk factors.
